# Chronic exposure to a predator or its scent does not inhibit male–male competition in male mice lacking brain serotonin

**DOI:** 10.3389/fnbeh.2014.00116

**Published:** 2014-04-08

**Authors:** Ying Huo, Qi Fang, Yao-Long Shi, Yao-Hua Zhang, Jian-Xu Zhang

**Affiliations:** ^1^State Key Laboratory of Integrated Management of Pest Insects and Rodents in Agriculture, Institute of Zoology, Chinese Academy of SciencesBeijing, China; ^2^Department of College of Life Sciences, University of Chinese Academy of SciencesBeijing, China

**Keywords:** predator, aggression, sexual attractiveness, central regulatory genes, serotonin, disinhibition

## Abstract

Although it is well-known that defective signaling of the 5-HT system in the brain and stressful stimuli can cause psychological disorders, their combined effects on male–male aggression and sexual attractiveness remain unknown. Our research aimed at examining such effects using tryptophan hydroxylase 2 (*Tph2*) knockout male mice vs. a rat- or rat scent-based chronic stress model. *Tph2^+/+^* and *Tph2^−/−^* male mice were placed individually into the rat home cage (rat), a cage containing soiled rat bedding (rat scent) or a cage containing fresh bedding (control) for 5 h every other day for 56 consecutive days. In *Tph2^+/+^* male mice, rat-exposure decreased male–male aggression and sexual attractiveness of urine odor relative to either rat scent-exposure or control; and rat scent-exposure decreased aggression rather than sexual attractiveness of urine odor compared with control. However, such dose-dependent and long-lasting behavioral inhibitory effects vanished in *Tph2^−/−^* male mice. RT-PCR assay further revealed that putative regulatory genes, such as AR, ERα and GluR4 in the prefrontal cortex, and TrkB-Tc and 5-HTR1A in the hippocampus, were down-regulated at the mRNA level in either rat- or rat scent-exposed *Tph2^+/+^* male mice, but partially in the *Tph2^−/−^* ones. Hence, we suggest that the dose-dependent and long-lasting inhibitory effects of chronic predator exposure on male–male aggression, sexual attractiveness of urine odor, and mRNA expression of central regulatory genes might be mediated through the 5-HT system in the brain of male mice.

## Introduction

Rodents rely heavily on chemical senses to detect potential predators and minimize predation risk in natural habitats (Herman and Valone, 2000; Dielenberg and McGregor, 2001; Zhang et al., 2008). The presence of predators or their chemical cues often have non-lethal, but negative, effects on the behavioral, and neurophysiological states of rodents (Dielenberg and McGregor, 2001; Zhang et al., 2003; Adamec et al., 2004; Apfelbach et al., 2005). For example, chronic exposure of rodents to a predator or its chemical cues can enhance anxiety-like behaviors and decrease aggression levels (Francis, 1988; Blanchard et al., 2001; Zhang et al., 2003). Rodents vs. predator-based paradigms have been used extensively to study innate fear- and stress-related psychiatric diseases, such as post-traumatic stress disorder (PTSD) (Hendrie et al., 1996; Adamec et al., 2006a).

Predator and predator scent are evidenced to be graded stress to rodents (Adamec et al., 2008). Unlike the strong stress resulting from close contact with the predator, mild predator scent presumably contains only the pheromones comprised of both volatile and non-volatile molecules from urine and sebaceous gland secretion, and odors emitted from the non-present predator (Kelliher et al., 1999). Previous studies have found that the effect of predator scent-exposure on rodent anxiety and risk assessment fall between controls and those exposed to a predator (Adamec et al., 2004, 2006b). Characterized by mouse-killing behavior (muricide), the rat is often regarded as a mouse predator (Molina et al., 1987; Beekman et al., 2005). Hence, rat- and rat scent-exposure are graded predator stress to mice.

Generally, stress would increase hypothalamic–pituitary–adrenal (HPA) functioning and provoke hyperactivity of the neuropeptide-secreting systems, which eventually lead to the release of stress hormones (Creel, 2001; Beekman et al., 2005). The elevated stress hormones alter metabolic pathways, which exert profound and diffusive effects, such as on reproduction competition ability, including aggressive and defensive levels, pheromone production, sexual attractiveness of urine odor, and related modulations of the neural systems (Creel, 2001; Sands and Creel, 2004; de Kloet et al., 2005; Zhang et al., 2008). To rodents, the released stress hormones induced by predator stimuli would lead to a state that presents reduced aggression levels, as well as less attractiveness to female conspecifics, which might weaken male–male competition, resulting in a reduction of reproductive success (Francis, 1988; Creel, 2001; Zhang et al., 2003).

As one of the most important neurotransmitters, serotonin (5-HT) exerts an extremely wide-ranging influence on rodents, including reproductive activity, aggression, sensory processing, stress adaption, behavioral disinhibition, cognition, memory, and emotion (Canli and Lesch, 2007; Tops et al., 2009; Liu et al., 2011; Kane et al., 2012; Sachs et al., 2013). Tryptophan hydroxylase 2 (Tph2) is required for the synthesis of central 5-HT. *Tph2* knockout mice were recently generated and have been used as a model animal to study 5-HT functions in the brain. *Tph2^−/−^* mice are characterized by only minute amounts of brain 5-HT, but normal serotonergic neurons (Liu et al., 2011; Gutknecht et al., 2012). *Tph2^−/−^* male mice display social impairments, communication deficits, and behavioral disinhibition indicated by impulsivity (Liu et al., 2011; Angoa-Pérez et al., 2012; Kane et al., 2012). They also show increased aggression in the resident–intruder paradigm and decreased anxiety-like behaviors in the elevated plus-maze (Mosienko et al., 2012). In spite of the 50% decrease in *Tph2* transcriptional activity, *Tph2^+/−^* mice only have a 10% reduction of brain 5-HT, which is insufficient to differentiate them from the *Tph2^+/+^* ones on aggression and anxiety-like behaviors (Gutknecht et al., 2012; Mosienko et al., 2012). Currently, little information is available about the modulation effect of 5-HT on mouse urine pheromone; however, our lab has found that *Tph2* knockout would increase the absolute levels of 2-heptanone and *E*-5-hepten-2-one; whereas, it decreases (s)-2-s-butyl-4,5-dihydrothiazole in male urine, indicative of the possible role of central 5-HT in the regulation of pheromone composition (unpublished data).

Previous studies have suggested that the effect of predator threat on rodents may be influenced by the central 5-HT system (Adamec et al., 2006a). In this context, the impulsive *Tph2^−/−^* male mice characterized by behavioral disinhibition can be expected to show less stress responses through modulations in the neural circuitry, which may play a causative role in the alterations of aggression levels and urine metabolites (Angoa-Pérez et al., 2012; Mosienko et al., 2012).

Many brain regions are known to play a role in the central processing of stressful stimuli from predators, such as the hippocampus, prefrontal cortex, lateral septum (LAS), and central amygdala (Hayley et al., 2001; Beekman et al., 2005; Joels et al., 2007). Stress hormones feed back to the brain and bind to two types of nuclear receptors serving as transcriptional regulators of brain region-specific susceptible effectors, such as 5-HT receptors, brain-derived neurotrophic factor (BDNF), BDNF receptor TrkB, AMPA (α-amino-3-hydroxy-5-methyl-4 isoxazole propionic acid) glutamate receptor (GluR) subunit, and the receptors of sex steroids that underlie stress-induced responses and behavioral adaptation, all of which function through the 5-HT system in the brain (Nibuya et al., 1999; Shutoh et al., 2000; Nelson and Chiavegatto, 2001; de Kloet et al., 2005; Lund et al., 2006; Nelson and Trainor, 2007). Sex steroid receptors in many brain regions have been suggested to play the modulation role of regulating neuroendocrine stress response and stress-related behaviors such as aggression via effects downstream on sites 5-HTR1A and 5-HTR1B (Simon et al., 1998; Handa and Weiser, 2013). Generally, androgen receptor (AR) and estrogen receptor α (ERα) are positively correlated with aggression levels (Nelson and Chiavegatto, 2001; Li et al., 2004; Scordalakes and Rissman, 2004). 5-HT also can change the expression of AMPA receptor subunits to alter the glutamatergic system activities and, thus, other neuronal functions (Shutoh et al., 2000). For example, GluR4 in the medial prefrontal cortex are in bidirectional control of social dominance hierarchy (Wang et al., 2011). Through 5-HT receptors, particularly 1A and 2A, stress decreases BDNF and increases full-length TrkB (TrkB-FL) expression, respectively, in the hippocampus to modulate learning, adaptive processes, inhibition of aggression, and anxiety- and depression-like behaviors in rodents (Nibuya et al., 1999; Vaidya et al., 2001; Pizarro et al., 2004; Kozlovsky et al., 2007; Martinowich and Lu, 2007; Ito et al., 2011). Unlike TrkB-FL acting as a reverse retrieve for BDNF, TrkB-Tc is speculated to be a negative regulator of TrkB-FL (Eide et al., 1996; Nibuya et al., 1999). Furthermore, 5-HTR1A and 5-HTR1B in the brain, especially in the hippocampus, are closely related to anxiety, aggression, and behavioral disinhibition (de Boer and Koolhaas, 2005; Ambar and Chiavegatto, 2009; Wang et al., 2009).

In rodents, even though there is little known about the central regulation of the urine metabolites, stress would inhibit aggression primarily via inhibitory inputs from the frontal cortex and hippocampus; whereas, the brain areas of medial amygdala (MEA), LAS, bed nucleus of the stria terminalis (BNST), and anterior hypothalamic area (AHA) have been evidenced to prompt the periaquaductal gray (PAG) into promoting aggression (Nelson and Trainor, 2007). In spite of many molecules, such as neurotransmitters, hormones, cytokines, enzymes, growth factors, and signaling molecular affect aggression, 5-HT remains the primary molecular determinant that others may act through the 5-HT signaling system (Nelson and Chiavegatto, 2001).

5-HIAA, the 5-HT metabolite, was found to be elevated only in the hippocampus and prefrontal cortex of both Balb/c and C57BL/6J mice when they were killed 20 min after rat exposure, which might suggest that the hippocampus and prefrontal cortex appear to be regulation sites of the central serotonin system on the effects of predator threat, especially on aggressive behaviors (Hayley et al., 2001). Accordingly, in the current study, prefrontal cortex-specific AR, ERα, and GluR4, and hippocampus-specific AR, ERα, BDNF, TrkB-Tc, 5-HTR1A, and 5-HTR1B that might participate in the stress-induced central responses which function through the 5-HT signaling system in the brain would be selected as the candidate neural substrates.

Previous studies have largely focused on the negative impacts of chronic predator exposure and the modulation roles of the 5-HT system (Apfelbach et al., 2005; Zhou et al., 2008). However, whether the impacts of chronic predator exposure on male–male competition are influenced in the absence of brain 5-HT and possible neural substrates remain unknown. In this study, we used *Tph2^+/+^* or *Tph2^−/−^* male mice vs. a rat- or rat scent-based paradigms to obtain insight into the differentiation between the two genotypes on the aggressive and defensive levels in staged male–male encounters and sexual attractiveness of urine odor after chronic predator exposure. Then, the mRNA expression of candidate genes in the prefrontal cortex and hippocampus were investigated to explore the possible neural substrates.

## Materials and methods

### Experimental animals

Twenty-four male Sprague–Dawley (SD) rats at 8 weeks of age were purchased from the Vital River Laboratories, Beijing, China, and acclimated for 4 weeks prior to use. The rats were housed individually in plastic cages (37 × 26 × 17 cm).

The *Tph2* line was born in our laboratory, while the parental lines were a kind gift from Dr. Yi Rao's laboratory (Peking University, Beijing, China). Mice were weaned at 21 days of age. Then, the male mice were housed in groups with their brothers up to 8 weeks old. After this, male mice were kept singly in plastic cages (27 × 12 × 17 cm). Female mice were always housed in groups of 4 per cage (27 × 12 × 17 cm).

The housing room was under a reversed 14L: 10D light/dark photoperiod (lights on at 7:00 pm), and the temperature was maintained at 23 ± 2°C. Food (standard mouse chow) and water were provided *ad libitum*. The animal maintenance and handling complied with the Institutional Guidelines for Animal Use and Care at the Institute of Zoology, Chinese Academy of Sciences. Ethics approval was obtained from the Institutional Ethics Committee of the Institute of Zoology, Chinese Academy of Sciences (approval number IOZ12017).

The *Tph2* line was maintained using crossing heterozygotes that the littermates contained wild-type, heterozygotes, and homozygous knockout mice. Genomic DNA was isolated from mouse tails using the phenol/chloroform extracting method at the age of 3 weeks for mouse genotyping. The primers for genotyping were: GCAGCCAGTAGACGTCTCTTAC; GGGCATCTCAGGACGTAGTAG; and GGGCCTGCCGATAGTAACAC. The thermal cycling conditions were as follows: 94°C for 5 min followed by 35 cycles of 94°C for 1 min, 62°C for 30 s, and 72°C for 1 min 30 s; then 72°C for 10 min (Liu et al., 2011).

### Procedures of rat- and rat scent- exposure

Twenty-four *Tph2^+/+^* or *Tph2^−/−^* male mice aged 12 weeks were randomly assigned into 3 groups of 8 mice each: rat group (each mouse was put into the rat home cage (37 × 26 × 17 cm) where the rat was individually housed and the bedding (250 g) was changed every week, and no barriers existed between the mouse and rat during rat exposure); rat scent group (each mouse was put into a cage (37 × 26 × 17 cm) containing 250 g soiled rat bedding that was used for 1 week by one rat); control group (each mouse was put into a cage (37 × 26 × 17 cm) that contained 250 g fresh bedding). Each *Tph2^+/+^* or *Tph2^−/−^* male mouse was regularly put into the corresponding standard plastic cage from 9:00 am to 2:00 pm, every other day for 56 consecutive days in a fixed donor-acceptor manner. Such treatments were alternately imposed on *Tph2^+/+^* or *Tph2^−/−^* male mice. In these rat-exposure pairings, the responses of rats to the mice ranged from watching, sniffing, and chasing with attacks. One assaultive rat exhibiting muricide was excluded.

Additionally, another 8 raw *Tph2^+/+^* male mice and 6 raw *Tph2^−/−^* male mice without any treatment (including exposure procedures and the following urine collection and behavioral tests presented below) were maintained in parallel for the comparison of gene expression between the two genotypes in the raw state.

### Urine collection

On days 57–59, the next 3 days after completing the 56 consecutive treatment days, the urine of all of the male mice subjected to the exposure procedures were collected in the dark phase, as we previously described (Zhang et al., 2007, 2008). In brief, each donor mouse was placed in a clean mouse cage (27 × 12 × 17 cm) with a wire grid 1 cm above the bottom. Once the animal urinated, the urine was immediately absorbed and transferred to an eppendorf tube in ice, using a disposable glass capillary (i.d. 1.8 mm, 15 cm long). Urine that was deposited with or next to feces was not collected. Urine was individually sealed and kept at −20°C until use.

### Encountering test

On days 60–62, the next 3 days after urine collection, male–male encountering tests were performed between male mice subjected to different exposure procedures within the same genotype, as previously described (Clancy et al., 1984; Zhang et al., 2008). Weight-matched male mice from different groups within the same genotype were paired and simultaneously placed into a clean mouse cage (27 × 12 × 17 cm) for 10 min continuous recording after initial aggressive behavior or defensive behavior. Such records were conducted by hand on a data sheet with a pre-calibrated time scale in units of 10 s using a stopwatch. A behavior pattern that lasted 10 s or less was treated as one unit. Each mouse was used only once a day. Aggressive behaviors included tail rattles, sideway postures, pushing, chasing, and biting; defensive behaviors included fleeing and upright postures.

### Binary test of urinary attractiveness

Binary choice tests via two capillaries were conducted to explore urinary attractiveness, as we previously described (Zhang et al., 2007, 2008). In brief, 14–15 adult female *Tph2^+/+^* mice in estrus (6–9 months of age) were used as test recipient subjects, each of which was used only once per day. The estrous status of female mice was determined by microscopic examination of vaginal epithelium. *Tph2^+/+^* female mice were given 1 day of rest between each test. Urine samples from different groups were randomly paired and presented to female mice by two identical disposable glass capillaries (internal diameter = 1.8 mm, length = 15 cm), which contained 2 μ L urine about 1 cm from the sample-containing end, and the other end sealed by odorless gum. The sample-containing end was presented to the test mice in their home cages simultaneously and kept approximately 2 cm apart from each other. We recorded investigating behavior (sniff within 1 cm of the tips or lick the end of the capillaries) for 3 min after the initial sniffing response. The durations that the test mouse spent investigating each odor were recorded using two hand-held stopwatches.

### Tissue sampling

On day 70, after 1 week of rest after the encountering tests, all of the male mice, including the ones subjected to the exposure procedure, urine collection and behavioral tests, and the raw ones without any treatment were directly and quickly (within 3 s) decapitated using a pair of sterile operating scissors. The prefrontal cortex and hippocampus were dissected in a mouse brain matrix on ice for the following real-time PCR analysis. The whole process was conducted in a separate dim room in the dark phase. The tissues were frozen in liquid nitrogen immediately and stored at −80°C until use.

### Real-time PCR

Total RNA of the prefrontal cortex and unilateral hippocampus were isolated using Trizol (Invitrogen), and cDNA was reverse-transcribed from total RNA (2 μ g) using PrimeScript® RT reagent Kit With gDNA Eraser (Perfect Real Time) (Takara), following the manufacturer's instructions. Real-time PCR was performed using the RealMasterMix (SYBR Green) (Tiangen). Specific primers were designed for genes, while the housekeeping gene β-actin was chosen as a control for normalizing the relative mRNA level (the primer sequences are available upon request). Twenty μL reaction agents were carried out in the Mx3005P quantitative PCR system (Stratagene, La Jolla, CA, USA) comprised of 9 μL of 2.5 × RealMasterMix/20 × SYBR solution, 1 μL of template cDNA, 0.5 μM of each primer, and 9 μL sterile water. Negative controls containing no template were also performed for each primer pair. The thermal cycling conditions were as follows: 95°C for 2 min followed by 40 cycles of 95°C for 20 s, 60°C for 20 s, and 68°C for 40 s. The melting curve analysis was performed to eliminate the presence of unspecific products by a high-resolution data collection during an incremental temperature change from 55 to 95°C with a ramp rate of 0.2°C/s. The data derived from the Mx3005P quantitative software were calculated using the 2^−△*C_T_*^ formula (Livak and Schmittgen, 2001; Wang et al., 2006).

### Statistical analysis

Behavioral data were analyzed by the Wilcoxon matched-pairs signed-rank test because of the non-normal distribution characteristics (the normality was checked using the Kolmogorov–Smirnov test). The comparisons of gene expression among the three groups of *Tph2^+/+^* male mice or the *Tph2^−/−^* ones that were subjected to exposure procedures, and the following urine collection and behavioral tests were analyzed by One-Way analysis of variance (ANOVA) with least significant difference (LSD) tests. The comparisons of gene expression between the raw *Tph2^+/+^* male mice and the raw *Tph2^−/−^* ones were analyzed by independent-samples *t*-test. All statistical analyses were conducted using SPSS 16.0 software (SPSS Inc., Chicago, IL, USA) with the critical value of α = 0.05.

## Results

### Rat- and rat scent-exposure inhibited male–male aggression in *Tph2^+/+^* male mice, but not in the *Tph2^−/−^* ones

**Figure 1:**
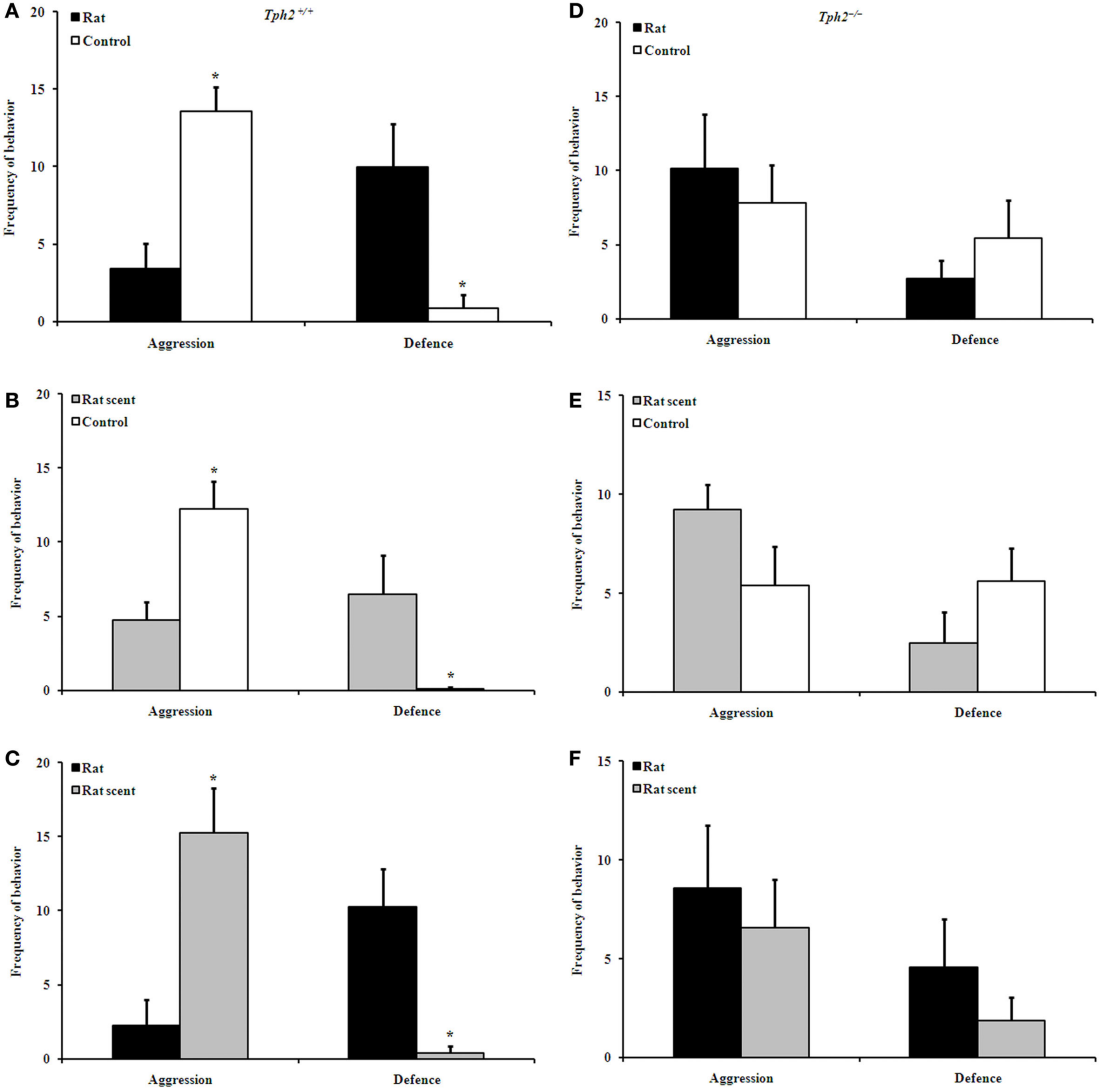
**Comparison of behavioral patterns between groups in the encountering test**. Aggression and defence of *Tph2*^+/+^ rat vs. *Tph2*^+/+^ control **(A)**, *Tph2*^+/+^ rat scent vs. *Tph2*^+/+^ control **(B)**, *Tph2*^+/+^ rat vs. *Tph2*^+/+^ rat scent **(C)**, *Tph2*^−/−^ rat vs. *Tph2*^−/−^ control **(D)**, *Tph2*^−/−^ rat scent vs. *Tph2*^−/−^ control **(E)**, *Tph2*^−/−^ rat vs. *Tph2*^−/−^ rat scent **(F)**. Mean ± SE, *n*_Rat_ = 7, *n*_Rat scent_ = 8, *n*_Control_ = 8, ^*^p < 0.05, Wilcoxon matched-pairs signed-rank test.

In staged dyadic encounters between *Tph2^+/+^* male mice, both the control [Figure 1A, Wilcoxon rank sum test, median 13.00 (interquartile range 6.000–18.00) vs. median 2.000 (interquartile range 0.000–12.00), *Z* = −2.201, *N* = 7, *P* = 0.028] and rat scent-exposed ones [Figure 1C, Wilcoxon rank sum test, median 16.00 (interquartile range 4.000–25.00) vs. median 0.000 (interquartile range 0.000–12.00), *Z* = −2.028, *N* = 7, *P* = 0.043] showed higher levels of aggression than the rat-exposed ones; and the control ones showed higher levels of aggression than the rat scent-exposed ones [Figure 1B, Wilcoxon rank sum test, median 12.50 (interquartile range 6.000–22.00) vs. median 6.500 (interquartile range 0.000–9.000), *Z* = −2.106, *N* = 8, *P* = 0.035].

Correspondingly, rat-exposed *Tph2^+/+^* males [Figure 1A, Wilcoxon rank sum test, median 13.00 (interquartile range 0.000–17.00) vs. median 0.000 (interquartile range 0.000–6.000), *Z* = −1.997, *N* = 7, *P* = 0.046] and the rat scent-exposed ones [Figure 1B, Wilcoxon rank sum test, median 4.500 (interquartile range 0.000–21.00) vs. median 0.000 (interquartile range 0.000–1.000), *Z* = −2.023, *N* = 8, *P* = 0.043] showed more defensive behaviors than the control ones; and the rat-exposed ones showed more defensive behaviors than the rat scent-exposed ones [Figure 1C, Wilcoxon rank sum test, median 12.00 (interquartile range 0.000–19.00) vs. median 0.000 (interquartile range 0.000–3.000), *Z* = −2.120, *N* = 7, *P* = 0.034].

However, such treatments did not differentiate aggressive and defensive behaviors in dyadic interactions between *Tph2^−/−^* male mice (Figures 1D–F).

### Rat-exposure decreased sexual attractiveness of urine odor in *Tph2^+/+^* male mice, but not in the *Tph2^−/−^* ones

**Figure 2:**
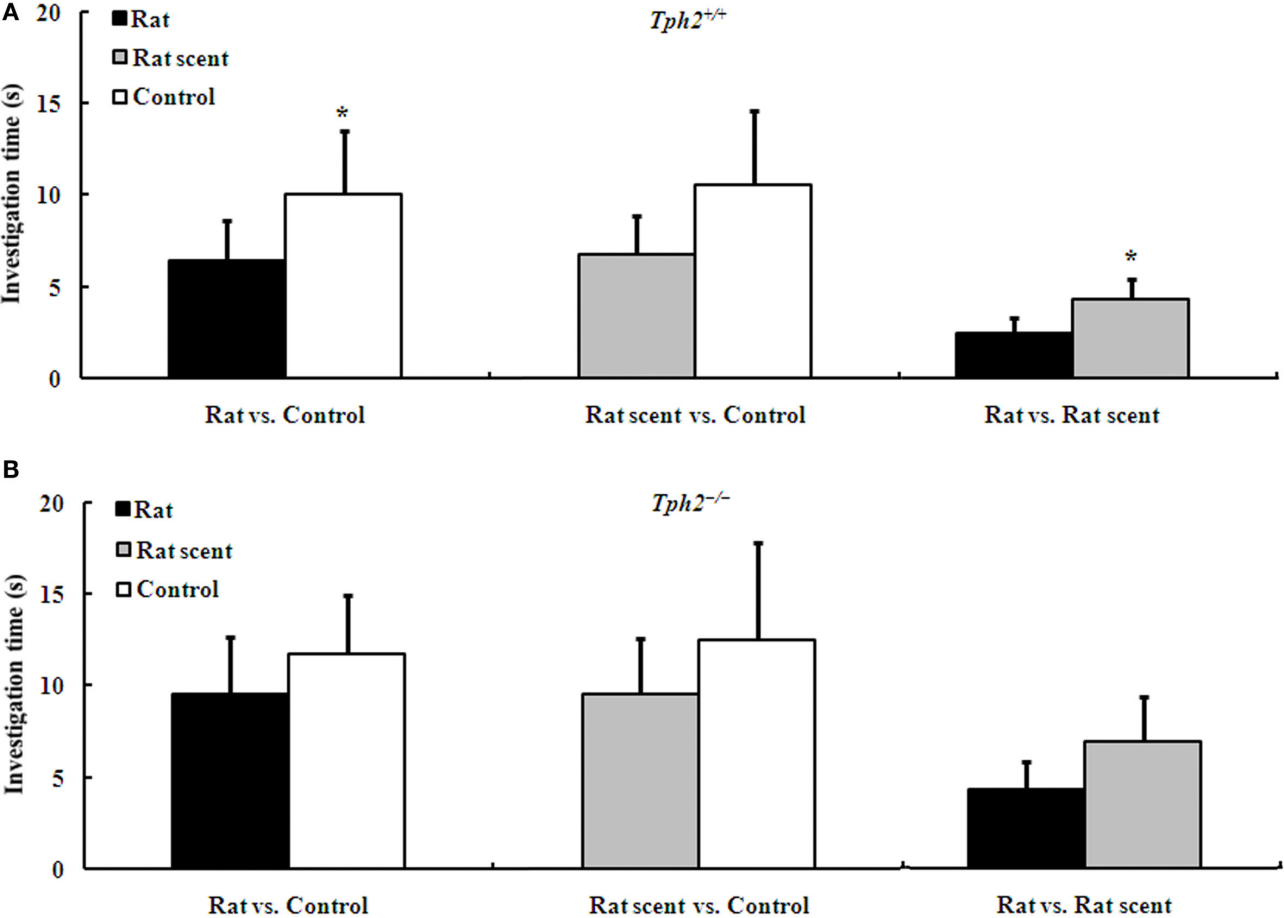
**Duration of the investigation (mean ± SE, *n*_Rat_ = 7, *n*_Rat scent_ = 8, *n*_Control_ = 8) of *Tph2^+/+^* female mice to male urine of different treatments during a 3-min choice test**. **(A)** Comparison of investigation time of *Tph2*^+/+^ female mice to the urine from two of the rat-exposed, rat scent-exposed and control *Tph2*^+/+^ male mice. **(B)** Comparison of investigation time of *Tph2*^+/+^ female mice to the urine from two of the rat-exposed, rat scent-exposed and control *Tph2*^−/−^ male mice. ^*^p < 0.05, Wilcoxon matched-pairs signed-rank test.

Two-choice tests unraveled that the urine odor from either rat scent-exposed *Tph2^+/+^* males [Figure 2A, Wilcoxon rank sum test, median 3.390 (interquartile range 0.000–15.76) vs. median 1.280 (interquartile range 0.000–12.25), *Z* = −2.073, *N* = 15, *P* = 0.038] or control *Tph2^+/+^* males [Figure 2A, Wilcoxon rank sum test, median 4.780 (interquartile range 0.400–44.87) vs. median 3.495 (interquartile range 0.000–30.79), *Z* = −2.103, *N* = 14, *P* = 0.035] induced higher attraction of *Tph2^+/+^* females than that from rat-exposed *Tph2^+/+^* males. However, *Tph2^+/+^* females showed no olfactory preferences between rat scent-exposed and control *Tph2^+/+^* males [Figure 2A, Wilcoxon rank sum test, median 3.140 (interquartile range 0.290–24.94) vs. median 3.585 (interquartile range 0.000–52.18), *Z* = −0.722, *N* = 14, *P* = 0.470].

Even so, two-choice tests revealed that the urine odor between any two groups of *Tph2^−/−^* males induced no apparent differences in attraction of *Tph2^+/+^* females (Figure 2B).

### Both rat- and rat scent-exposure down-regulated the gene expression in the prefrontal cortex of *Tph2^+/+^* male mice, but not in the *Tph2^−/−^* ones

**Figure 3:**
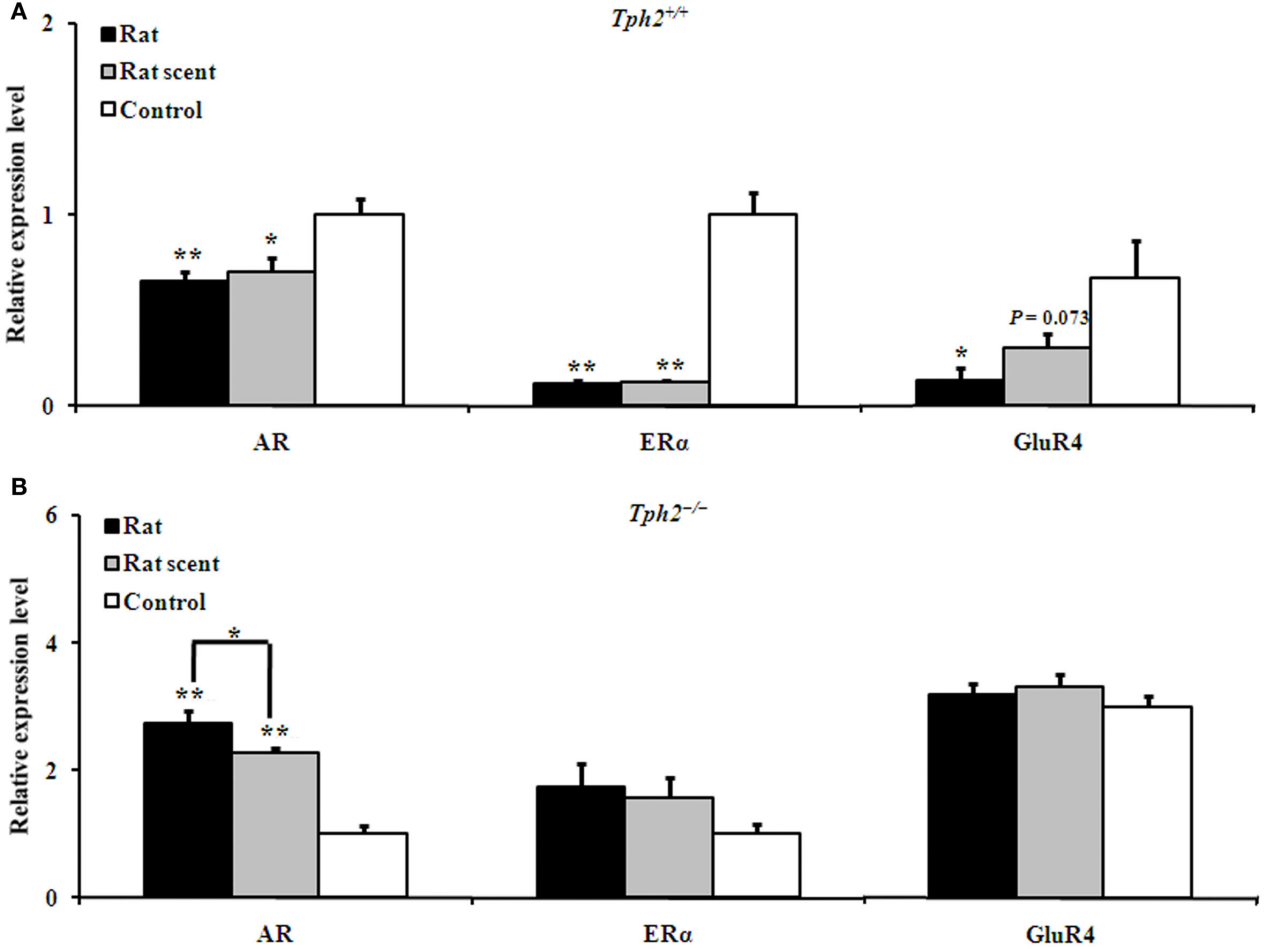
**Comparison of the relative expression of genes in the prefrontal cortex of different groups**. **(A)** Expression patterns of AR, ERα and GluR4 in the prefrontal cortex of the rat-exposed, rat scent-exposed and control *Tph2*^+/+^ male mice (mean ± SE, *n*_Rat_ = 4, *n*_Rat scent_ = 4, *n*_Control_ = 4). In order to obtain a large amount for each *Tph2*^+/+^ biological replicate, the prefrontal cortex from two male mice in the same group were ground together for RNA extraction. **(B)** Expression patterns of AR, ERα and GluR4 in the prefrontal cortex of the rat-exposed, rat scent-exposed and control *Tph2*^−/−^ male mice (mean ± SE, *n*_Rat_ = 7, *n*_Rat scent_ = 8, *n*_Control_ = 8). The data were analyzed by One-Way ANOVA, followed by LSD *post-hoc t-tests* (^*^P < 0.05; ^**^p < 0.01).

In the prefrontal cortex of *Tph2^+/+^* male mice, the expression of AR [Figure 3A, One-Way ANOVA, *F*_(2, 9)_ = 6.913, *P* = 0.015], ERα [Figure 3A, One-Way ANOVA, *F*_(2, 9)_ = 55.31, *P* = 0.000] and GluR4 [Figure 3A, One-Way ANOVA, *F*_(2, 9)_ = 4.594, *P* = 0.042] apparently differed among the 3 groups. Compared with the control ones, *Tph2^+/+^* AR (Figure 3A, LSD *post-hoc t-tests*, rat-exposure: *P* = 0.007; rat scent-exposure: *P* = 0.016), ERα (Figure 3A, LSD *post-hoc t-tests*, rat-exposure: *P* = 0.000; rat scent-exposure: *P* = 0.000) and GluR4 (Figure 3A, LSD *post-hoc t-tests*, rat-exposure: *P* = 0.016; rat scent-exposure: *P* = 0.073, marginal significance) expression were down-regulated.

However, unlike *Tph2^+/+^* male mice, the *Tph2^−/−^* ones up-regulated AR expression [Figure 3B, One-Way ANOVA, *F*_(2, 20)_ = 42.51, *P* = 0.000] in the rat- (Figure 3B, LSD *post-hoc t-tests*, *P* = 0.000) and rat scent-exposed male mice (Figure 3B, LSD *post-hoc t-tests*, *P* = 0.000) as compared with the control ones. In addition, AR was up-regulated in the rat-exposed *Tph2^−/−^* male mice more than the rat scent-exposed ones (Figure 3B, LSD *post-hoc t-tests*, *P* = 0.027). The expression of other genes, including ERα and GluR4 did not differ among the 3 groups of *Tph2^−/−^* male mice (Figure 3B).

**Figure 4:**
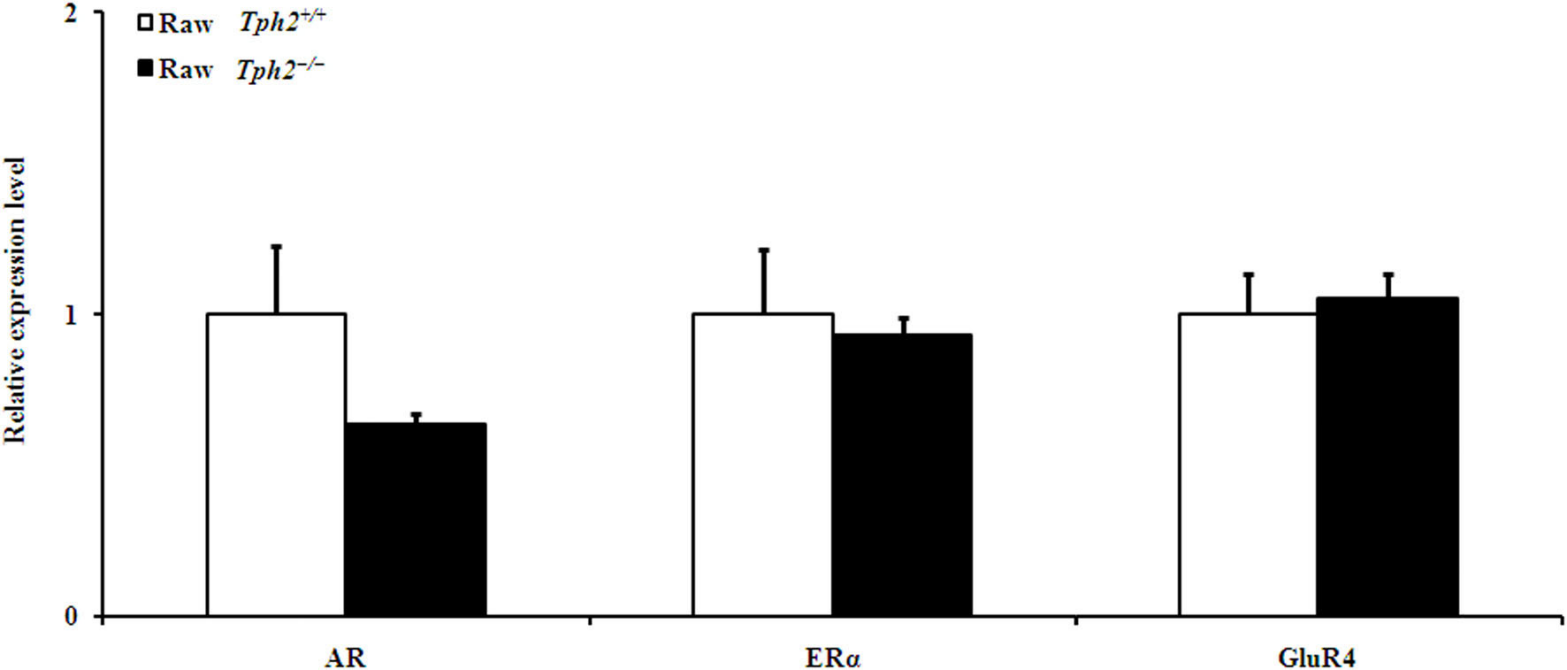
**Comparison of the relative expression of genes in the prefrontal cortex between the raw *Tph2^+/+^* male mice and the raw *Tph2^−/−^* ones (mean ± SE, for the raw *Tph2^+/+^* male mice, *n* = 8; for the raw *Tph2^−/−^* male mice, *n* = 6)**. The data were analyzed by independent-samples *t*-test, no significant differences were showed between groups.

Additionally, the expression of AR, ERα, and GluR4 in the prefrontal cortex did not differ between the raw *Tph2^+/+^* male mice and the raw *Tph2^−/−^* ones (Figure 4).

### Both rat- and rat scent-exposure down-regulated hippocampal gene expression more in *Tph2^+/+^* male mice than in the *Tph2^−/−^* ones

**Figure 5:**
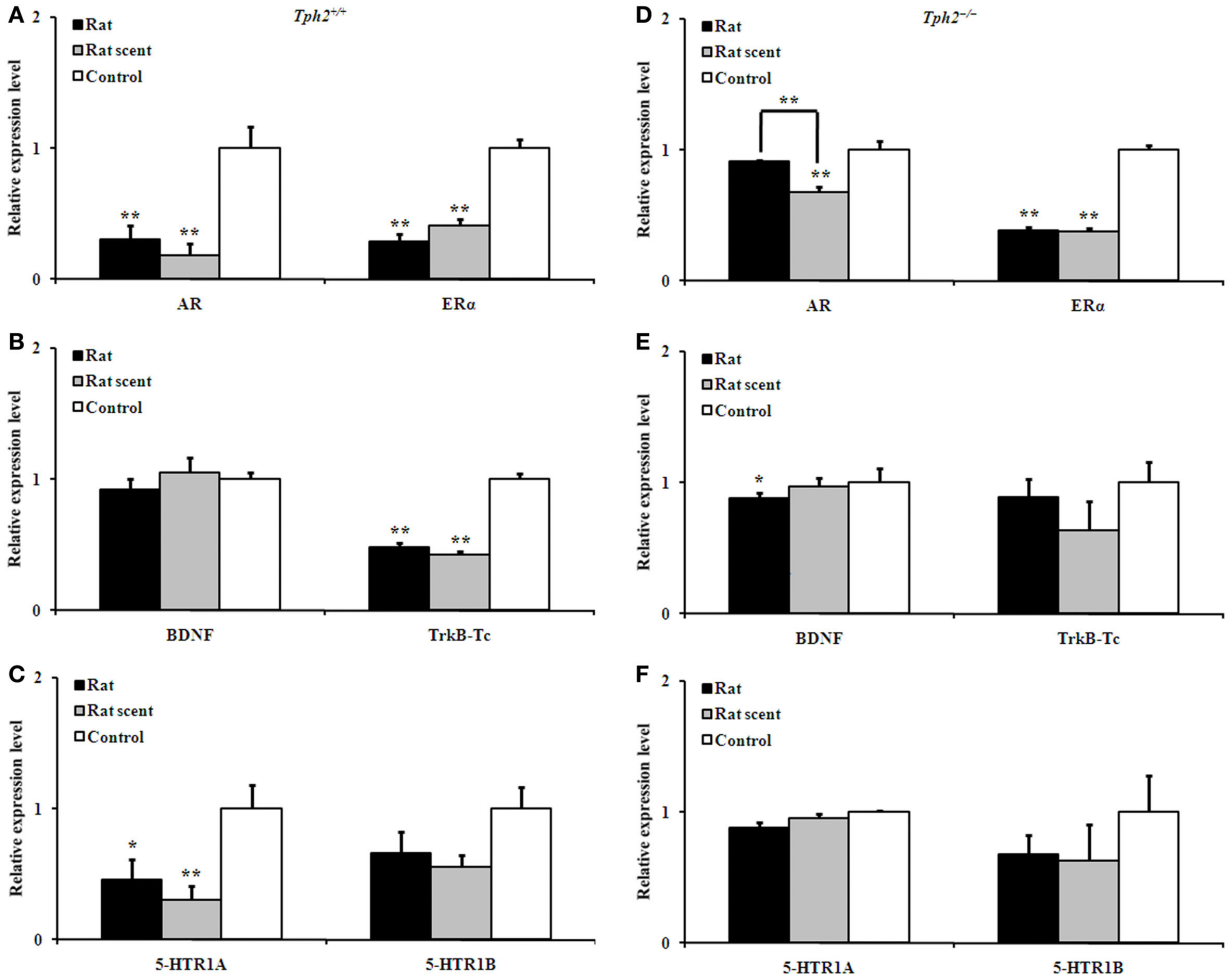
**Comparison of the relative expression of genes in the hippocampus of different groups**. Expression patterns of AR and ERα **(A)**, BDNF and TrkB-Tc **(B)**, and 5-HTR1A and 5-HTR1B **(C)** in the hippocampus of the rat-exposed, rat scent-exposed and control *Tph2*^+/+^ male mice. Expression patterns of AR and ERα **(D)**, BDNF and TrkB-Tc **(E)**, and 5-HTR1A and 5-HTR1B **(F)** in the hippocampus of the rat-exposed, rat scent-exposed and control *Tph2*^−/−^ male mice. Mean ± SE, *n*_Rat_ = 4, *n*_Rat scent_ = 4, *n*_Control_ = 4. In order to obtain a large amount for each biological replicate, the hippocampi from two male mice in the same group were ground together for RNA extraction. The data were analyzed by One-Way ANOVA, followed by LSD *post-hoc t-tests* (^*^P < 0.05; ^**^p < 0.01).

In the hippocampus of *Tph2^+/+^* male mice, the AR [Figure 5A, One-Way ANOVA, *F*_(2, 9)_ = 12.62, *P* = 0.002], ERα [Figure 5A, One-Way ANOVA, *F*_(2, 9)_ = 46.55, *P* = 0.000], TrkB-Tc [Figure 5B, One-Way ANOVA, *F*_(2, 9)_ = 75.98, *P* = 0.000] and 5-HTR1A [Figure 5C, One-Way ANOVA, *F*_(2, 9)_ = 5.875, *P* = 0.023] expression were significantly different among the 3 groups. As compared with the control ones, *Tph2^+/+^* hippocampal AR (Figure 5A, LSD *post-hoc t-tests*, rat group: *P* = 0.003; rat-scent group: *P* = 0.001), ERα (Figure 5A, LSD *post-hoc t-tests*, rat group: *P* = 0.000; rat-scent group: *P* = 0.000), TrkB-Tc (Figure 5B, LSD *post-hoc t-tests*, rat group: *P* = 0.000; rat-scent group: *P* = 0.000), and 5-HTR1A (Figure 5C, LSD *post-hoc t-tests*, rat group: *P* = 0.033; rat-scent group: *P* = 0.010) expression were down-regulated.

In the hippocampus of *Tph2^−/−^* male mice, the AR [Figure 5D, One-Way ANOVA, *F*_(2, 9)_ = 14.43, *P* = 0.002], ERα [Figure 5D, One-Way ANOVA, *F*_(2, 9)_ = 147.0, *P* = 0.000] and BDNF [Figure 5E, One-Way ANOVA, *F*_(2, 9)_ = 4.142, *P* = 0.053, marginal significance] expression also presented significant differentiation among the 3 groups. As compared with the control ones, *Tph2^−/−^* hippocampal ERα was both down-regulated in the mice exposed to either rat (Figure 5D, LSD *post-hoc t-tests*, *P* = 0.000) or rat scent (Figure 5D, LSD *post-hoc t-tests*, *P* = 0.000). However, *Tph2^−/−^* hippocampal AR of rat scent-exposed mice (Figure 5D, LSD *post-hoc t-tests*, *P* = 0.001) and BDNF of rat-exposed mice (Figure 5E, LSD *post-hoc t-tests*, *P* = 0.019) were only down-regulated relative to the control ones. Additionally, the strong rat-exposure stress increased *Tph2^−/−^* hippocampal AR expression than the milder rat scent-exposure stress (Figure 5D, LSD *post-hoc t-tests*, *P* = 0.004).

*Tph2^+/+^* BDNF, *Tph2^+/+^* 5-HTR1B, *Tph2^−/−^* TrkB-Tc, *Tph2^−/−^* 5-HTR1A, and *Tph2^−/−^* 5-HTR1B expression in the hippocampus were not changed by rat- or rat scent- exposure (Figures 5B,C,E,F).

**Figure 6:**
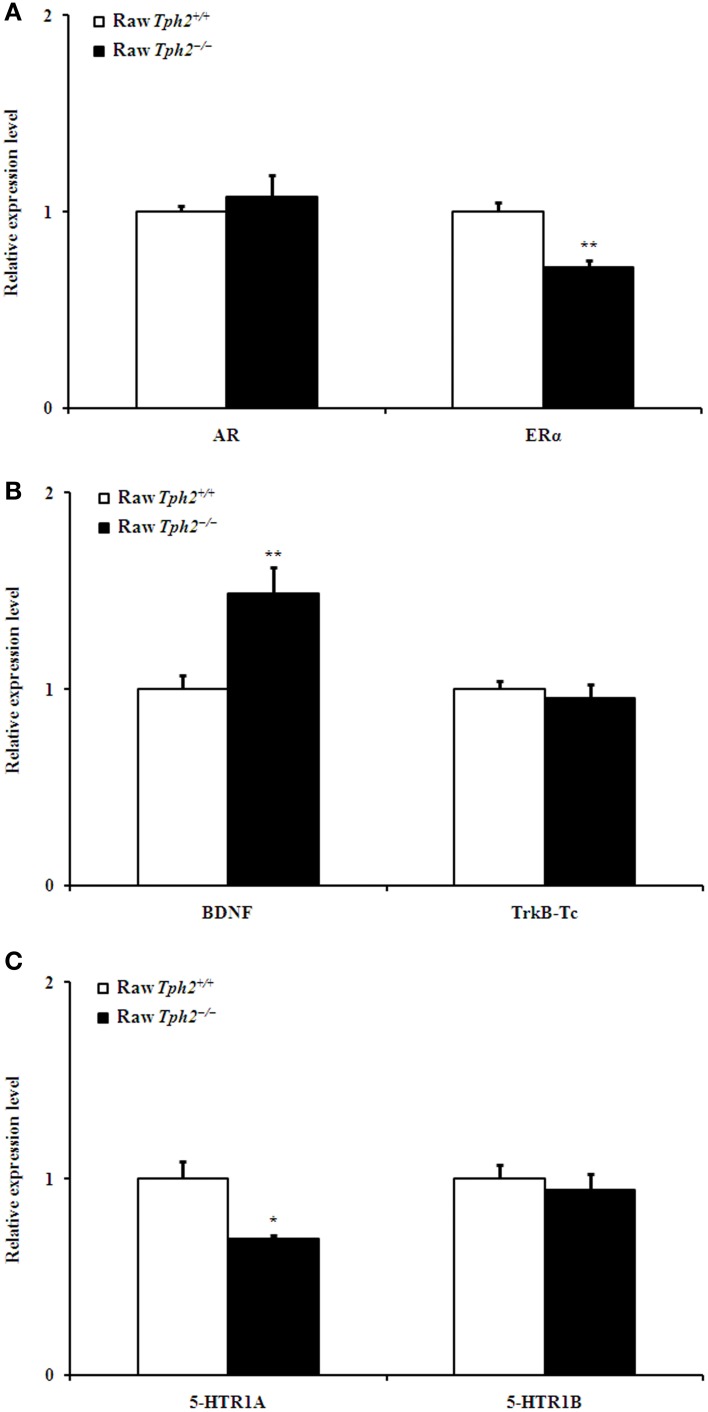
**Comparison of the relative expression of AR and ERα **(A)**, BDNF and TrkB-Tc **(B)**, and 5-HTR1A and 5-HTR1B **(C)** in the hippocampus between the raw *Tph2*^+/+^ male mice and the raw *Tph2*^−/−^ ones (mean ± SE, for the raw *Tph2^+/+^* male mice, *n* = 8; for the raw *Tph2^−/−^* male mice, *n* = 6)**. The data were analyzed by independent-samples *t*-test (^*^P < 0.05; ^**^p < 0.01).

However, compared with the raw *Tph2^+/+^* males, hippocampal ERα (Figure 6A, independent-samples *t*-test, *t* = 4.464, *df* = 12, *P* = 0.001) and 5-HTR1A (Figure 6C, independent-samples *t*-test, *t* = 2.888, *df* = 12, *P* = 0.014) expression of the raw *Tph2^−/−^* ones decreased; whereas, the BDNF (Figure 6B, independent-samples *t*-test, *t* = −3.571, *df* = 12, *P* = 0.004) expression increased. The expression of other genes, including AR, TrkB-Tc, and 5-HTR1B in the hippocampus did not differ between the two genotypes in the raw state (Figures 6A–C).

## Discussion

Our data showed that chronic exposure to rat or rat scent both could produce long-lasting inhibitory effects on *Tph2^+/+^* male mice, reflected in the down-regulated male–male aggression, sexual attractiveness of urine odor, and mRNA expression of central regulatory genes. However, such inhibitory effects were reduced or abolished in *Tph2^−/−^* male mice.

One of the primary responses to stress is an increase in the concentration of circulating adrenal glucocorticooids (GC). Instead of GC elevations lasting for hours, chronic exposure to a predator or its scent causes GC levels to remain high for more than a few days, causing a broad and long-lasting negative effects (Creel, 2001). Such impacts have been widely proven in rats and mice, focusing on anxiogenic effects as measured in the EPM, light/dark box, and acoustic startle tests (Dielenberg and McGregor, 2001; Adamec et al., 2006a). For example, the anxiety state of rats exposed to a cat lasted 21 days or more, similar to long-lasting effects on acoustic startle responses of CD-1 mice exposed to rat odor (Adamec and Shallow, 1993; Hebb et al., 2003a).

It is a general belief that psychological stressors, such as the threat of a predator, would negatively affect social and sexual behaviors, such as aggression levels and sexual attractiveness (Francis, 1988; Blanchard et al., 2001; Zhang et al., 2003; Apfelbach et al., 2005). Receptive females expend much more effort during reproduction than do males; hence, they prefer to select high-quality males, in order to increase the probability of reproductive success and offspring survival (Huck and Banks, 1982). Both aggression levels and urinary attractiveness to female conspecifics are representatively manifested in the ability of male mice in male–male competition. In our current study, the descending degree in aggression levels of *Tph2^+/+^* males, dependent upon stimulation intensity, strongly supported the dose-dependent negative influences and predicted competitive ability. Furthermore, such inhibition lasted for a minimum of 6 days, which is consistent with the proven long-lasting effects on anxiogenic states, as stated above.

However, rat scent-exposure did not reduce the urinary attractiveness of *Tph2^+/+^* male mice. Such an anti-intuitive result might be ascribed to the insufficient intensity of the rat scent used, since the intensity of predator stress has been suggested as an important factor in the severity of long-lasting effects (Hebb et al., 2003b). For instance, we previously reported that male mice exposed to a low dose of cat urine showed higher sexual attractiveness to female conspecifics than the control ones (Zhang et al., 2008). Even so, the results that urine odor from both rat scent-exposed and control *Tph2^+/+^* males were more attractive to females than the rat-exposed ones still demonstrated the dose-dependent inhibitory effects of chronic predator exposure on male–male competition.

Accordingly, male–male aggression and sexual attractiveness of urine odor of *Tph2^+/+^* male mice were long-lastingly inhibited by graded stress from rat and rat scent, depending on the stimulation intensity in the current study, which is consistent with and expands on previously published reports.

The pharmacological challenge of neuronal 5-HT, such as treatment with 5-HTR1A and 5-HTR1B agonists, depletion using *p*-Chlorophenylalanine (PCPA; irreversible inhibitor of the 5-HT synthesizing enzyme tryptophan hydroxylase), or intracerebral injection of the 5-HT neurotoxic agent 5,7-dihydroxytryptamine (5,7-DHT), also induces multiple alterations, such as aggression levels and hormone release (Willoughby et al., 1982; Chiavegatto et al., 2001; de Boer and Koolhaas, 2005). Even so, these manipulations have some limitations to consider, including time (stages of pre- or post-natal development), target tissue (brain or periphery), route, effective time, and complex mechanisms regulating 5-HT neuron firing, presynaptic release, and post-synaptic receptor expression (Miyazaki et al., 2011; van Kleef et al., 2012). However, genetic disruption in the *Tph2* affects the central 5-HT system throughout the lifespan, leading to possible alterations in the formation of the neural circuitry mediating emotion and stress adaption during a critical period of brain development (Adamec et al., 2006a; Liu et al., 2011).

Accordingly, in the current study, the predator or its scent exposure did not alter aggression and urinary attractiveness of *Tph2^−/−^* male mice, suggesting that brain 5HT deficiency might prevent the inhibitory effects by affecting sensory processing and, thus, induce behavioral disinhibition (Raleigh et al., 1991; Nelson and Chiavegatto, 2001; Freichel et al., 2004; Canli and Lesch, 2007; Tops et al., 2009; Liu et al., 2011; Kane et al., 2012). Moreover, the reduced expression of ERα and 5-HTR1A accompanied by increased BDNF in the hippocampus of the raw *Tph2^−/−^* male mice, which may suggest that deficiency of brain 5-HT synthesis likely extends beyond the serotonin system itself (Murphy et al., 2003; Adamec et al., 2006a).

The expression of the prefrontal cortex- and hippocampus-specific genes, which were selectively used in the current study, can reasonably reflect stress-induced central responses and elucidate the mechanisms underlying behavioral alterations. In the current study, the down-regulation of AR, ERα and GluR4 in the prefrontal cortex, and TrkB-Tc and 5-HTR1A in the hippocampus might result from stress responses and, in turn, provide the neural substrates for the alterations of male–male aggression and sexual attractiveness in the *Tph2^+/+^* male mice exposed to a rat or rat scent. The decreased expression of GluR4 in the prefrontal cortex and 5-HTR1A in the hippocampus of stressed *Tph2^+/+^* male mice was consistent with previous literature (Wang et al., 2009, 2011). Despite being in line with the expected low expression in the hippocampus of stressed *Tph2^+/+^* TrkB-Tc, the results of the indifferent *Tph2^+/+^* BDNF expression were surprising (Nibuya et al., 1999; Vaidya et al., 2001; Pizarro et al., 2004; Kozlovsky et al., 2007). Such bifurcations originated mostly from differences in genetic background, as previous studies on the impacts of predator stress on hippocampal BDNF expression focusing on rats other than the C57BL/6J × 129S5/S mice used in our study. Furthermore, the decreased hippocampal BDNF expression of C57BL/6 mice followed acute social stress other than chronic predator stress in our study. In this respect, it is necessary to note, instead of decreased hippocampal BDNF mRNA expression in rat vs. predator-based models, the reduced hippocampal TrkB-Tc mRNA expression might play an important role in modulation of stress response in mice vs. predator-based paradigms, according to our results. AR and ERα have been suggested to be positively correlated with aggression level, as stated above (Li et al., 2004; Scordalakes and Rissman, 2004). In this research, AR and ERα in the prefrontal cortex, rather than in the hippocampus, might contribute to central regulation and related behavioral alterations after predator stress, thus confirming and extending the aforementioned work.

In addition, although rat- and rat-scent exposure could have different effects on male–male competition of *Tph2^+/+^* male mice, their differences in threat intensity were not sufficient to differentiate the mRNA expression of the examined genes.

*Tph2^−/−^* male mice showed different mRNA expression patterns from the *Tph2^+/+^* ones. Specifically, rat- and/or rat scent- exposure up-regulated prefrontal AR expression, but down-regulated hippocampal AR, ERα, and BDNF expression, suggesting that 5-HT might mediate central regulation of responses to predation risk. However, such alterations of gene expression appeared to exert no impacts on male–male aggression and sexual attractiveness in *Tph2^−/−^* male mice, likely reflecting that these genes were not able to work through the defective 5-HT system (Nelson and Chiavegatto, 2001).

In conclusion, the current results are consistent with previous work that exposure to a predator or predator scent causes dose-dependent and long-lasting negative impacts on rodents. However, we particulalrly relate predator-based chronic stress to brian 5-HT-deficent male mice and male–male competition ability for potential female mates, as indicated by male–male aggression and urinary attractiveness to female conspecifics. For the first time, inhibitory effects on these behavioral measures are found to be abolished in *Tph2^−/−^* male mice. We also demonstrate that AR, ERα and GluR4 expression in the prefrontal cortex, and TrkB-Tc and 5-HTR1A expression in the hippocampus are changed in response to predation risk. In turn, they may regulate the alterations of male–male aggression and urinary attractiveness to female conspecifics through the 5-HT system in the brain of male mice.

### Conflict of interest statement

The authors declare that the research was conducted in the absence of any commercial or financial relationships that could be construed as a potential conflict of interest.
